# Evolution and dysfunction of human cognitive and social traits: A transcriptional regulation perspective

**DOI:** 10.1017/ehs.2022.42

**Published:** 2022-09-26

**Authors:** Roman Zug, Tobias Uller

**Affiliations:** Department of Biology, Lund University, Lund, Sweden

**Keywords:** transcriptional regulation, haploinsufficiency, loss of function, neurodevelopmental disorders, human self-domestication

## Abstract

Evolutionary changes in brain and craniofacial development have endowed humans with unique cognitive and social skills, but also predisposed us to debilitating disorders in which these traits are disrupted. What are the developmental genetic underpinnings that connect the adaptive evolution of our cognition and sociality with the persistence of mental disorders with severe negative fitness effects? We argue that loss of function of genes involved in transcriptional regulation represents a crucial link between the evolution and dysfunction of human cognitive and social traits. The argument is based on the haploinsufficiency of many transcriptional regulator genes, which makes them particularly sensitive to loss-of-function mutations. We discuss how human brain and craniofacial traits evolved through partial loss of function (i.e. reduced expression) of these genes, a perspective compatible with the idea of human self-domestication. Moreover, we explain why selection against loss-of-function variants supports the view that mutation-selection-drift, rather than balancing selection, underlies the persistence of psychiatric disorders. Finally, we discuss testable predictions.

## Graphical abstract

**Figure:**
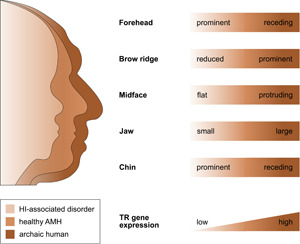

**Social media summary:** Loss of function of transcriptional regulator genes links evolution and dysfunction of human cognitive and social traits.

## Introduction

What sets us apart from other species is one of humanity's big questions. The most obvious candidate traits are related to human cognition and sociality, including problem solving, social cognition and communication (Laland & Seed, 2021). The extraordinary cognitive and social abilities that evolved in the human lineage are largely based on changes in brain development, structure and function (Sousa et al., 2017a; Chin et al., 2022), accompanied by changes in craniofacial development and morphology, which enabled the evolution of the human face with its unique possibilities for social interaction and communication (Wilkins, 2017; Lacruz et al., 2019).

However, this remarkable success story also has a dark side. The genetic and developmental changes that enabled the evolution of human cognitive and social traits appear also to be associated with the risk of debilitating disorders in which these traits are disrupted, such as intellectual disability, schizophrenia and autism (Doan et al., 2018; Pattabiraman et al., 2020). Given that natural selection has been so powerful in shaping our unique cognition and sociality, why has it been unable to eliminate the genetic variants that predispose to the associated mental disorders with severe fitness costs?

Here we argue that loss of function (LOF) of genes involved in transcriptional regulation (TR) of brain and face development represents a crucial link between the adaptive evolution and the dysfunction of human cognitive and social traits. The argument is based on a crucial property of TR genes, namely their haploinsufficiency, which makes them particularly sensitive to LOF mutations. After a short overview of human disorders associated with brain and face development (neurodevelopmental disorders and neurocristopathies), we show that many of them are caused by LOF of haploinsufficient TR genes. We then discuss evidence showing that human brain and craniofacial traits evolved owing to partial LOF (reduced expression) of TR genes, and we show that this perspective is compatible with an evolutionary scenario of human self-domestication, including a key role of the neural crest. Further, we explain why purifying selection against LOF variants in TR genes makes mutation-selection-drift a more likely explanation than balancing selection for the evolutionary persistence of mental disorders. Finally, we list predictions from this theory that can be tested using powerful experimental model systems such as brain organoids, and comparative genomics involving ancient humans, non-human primates and domesticated species.

## Human brain and face development and associated disorders

Human brain and face development is governed by complex processes involving cell proliferation, migration and differentiation. These processes have been covered comprehensively in excellent reviews of brain (Taverna et al., 2014; Molnár et al., 2019; Kelley & Paşca, 2022) and craniofacial development (Minoux & Rijli, 2010; Cordero et al., 2011; Murillo-Rincón & Kaucka, 2020). Moreover, brain and craniofacial development are intimately connected (Marcucio et al., 2015; LaMantia, 2020; Naqvi et al., 2021) and tightly linked to the neural crest (NC), a transient embryonic cell population. Neural crest cells actively migrate throughout the developing embryo and differentiate into a large number of cell types and tissues. For example, cranial NC cells give rise to craniofacial cartilage and bones, thus forming large parts of the head and face (Minoux & Rijli, 2010; Cordero et al., 2011; Murillo-Rincón & Kaucka, 2020).

The cellular processes that underlie brain and face development are orchestrated by gene regulatory networks (GRNs). In recent years, there has been great progress in elucidating the genetics and GRNs underlying brain (Nord et al., 2015; Trevino et al., 2021) and craniofacial development (White et al., 2021; Naqvi et al., 2022). Key players in these GRNs are transcription factors (TFs) that bind to cell type-specific *cis-*regulatory elements (CREs), in particular enhancers, thus driving gene expression programmes that control cell fate determination, migration, and maturation. Cell type-specific gene expression is assisted by transcriptional cofactors and additional regulatory proteins involved in chromatin remodelling and DNA methylation. Here, we subsume the action of TFs, cofactors, chromatin remodellers and other proteins that regulate cell type-specific gene expression under the term transcriptional regulation (TR).

Disruption of the GRNs underlying brain and face development can lead to a plethora of disorders and syndromes. Those that result from disrupted brain development are called neurodevelopmental disorders (NDDs). These include intellectual disability (ID), autism spectrum disorders (ASD), schizophrenia (SCZ), epilepsy, attention deficit hyperactivity disorder (ADHD), bipolar disorder (BD), major depressive disorder (MDD) and speech and language disorders (for references, see Table 1 in Zug, 2022).

Disorders arising from defects in NC cell specification and migration are called neurocristopathies (NCPs; Vega-Lopez et al., 2018; Sato et al., 2019). Neurocristopathies are highly diverse and affect disparate tissues; here we focus on those that involve the cranial NC and hence result in craniofacial abnormalities. Given the tight integration of brain and craniofacial development, many human disorders and syndromes show features of both NDDs and NCPs.

## Many disorders of brain and face development are caused by loss of function of haploinsufficient TR genes

In the past 10 years or so, our understanding of the genetic architecture of NDDs such as ASD and SCZ has improved considerably. It has become clear that, despite high genetic heterogeneity, NDD risk genes converge on shared functional pathways, such as synaptic function and TR (Moyses-Oliveira et al., 2020; Parenti et al., 2020; Mossink et al., 2021). Hence, there are many NDDs that are mainly caused by rare, large-effect variants in TR genes (modified by the polygenic background of common variants of small effect). For example, TR genes associated with ASD risk include *ARID1B*, *BCL11A*, *CHD8*, *FOXP1*, *KMT2C*, *MBD5*, *POGZ*, *TBR1*, *TCF4* and *TCF20* (Sestan & State, 2018; Iakoucheva et al., 2019; Sullivan et al., 2019; Lord et al., 2020; Satterstrom et al., 2020; Zug, 2022). TR genes associated with SCZ risk include *AUTS2*, *CHD4*, *CHD8*, *EP300*, *KDM2B*, *KMT2C*, *KMT2D*, *SETD1A*, *SMARCC2* and *TCF4* (Girard et al., 2011; McCarthy et al., 2014; Jia et al., 2018; Doostparast Torshizi et al., 2019; Howrigan et al., 2020).

**Figure 1.**
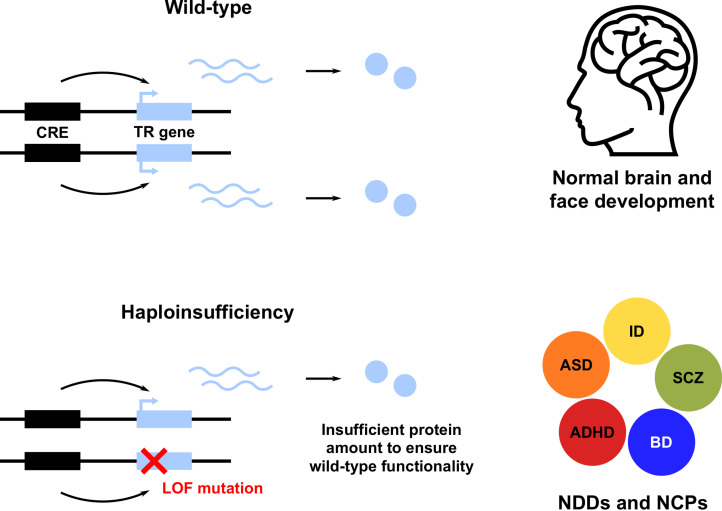
Heterozygous loss of function (LOF) of haploinsufficient genes involved in transcriptional regulation (TR) of brain and face development leads to neurodevelopmental disorders (NDDs) and neurocristopathies (NCPs). Only a subset of NDDs are shown, including attention deficit hyperactivity disorder (ADHD), autism spectrum disorder (ASD), bipolar disorder (BD), intellectual disability (ID) and schizophrenia (SCZ). CRE, *cis-*regulatory element.

A striking feature of these and many other TR genes is their haploinsufficiency (HI): a single functional allele is insufficient to sustain normal gene function; in other words, these genes do not tolerate heterozygous LOF (Figure 1; see Boxes 1 and 2; Zug, 2022). Note that we use the term HI in a broader sense, describing both the property of genes intolerant to heterozygous LOF and the resulting condition. Owing to the HI of many TR genes, NDD risk variants affecting these genes are predominantly rare inherited or *de novo* heterozygous LOF mutations. This has been shown for ASD (De Rubeis et al., 2014; Iossifov et al., 2014; Krumm et al., 2015; Ji et al., 2016; Sestan & State, 2018; Sullivan et al., 2019), SCZ (Girard et al., 2011; Gulsuner et al., 2013; McCarthy et al., 2014; Pardiñas et al., 2018; Howrigan et al., 2020) and other NDDs (Samocha et al., 2014; Kataoka et al., 2016; Deciphering Developmental Disorders Study, 2017; Kosmicki et al., 2017; Brunet et al., 2021; Wainberg et al., 2022). Furthermore, NDD risk variants also affect non-coding CREs, resulting in regulatory LOF, such as enhancer deletion, disruption and disconnection (see Box 2; D'haene & Vergult, 2021). Frequently, affected CREs regulate those TR genes that are themselves associated with disease risk (Short et al., 2018; Turner & Eichler, 2019; Zhou et al., 2019). These findings demonstrate that heterozygous LOF of haploinsufficient TR genes is an important cause of NDDs. A more detailed account of NDDs caused by HI of TR genes is given in Table S1.

Box 1: Why are so many TR genes haploinsufficient?

It has long been known that many NDDs, NCPs and other developmental disorders are due to HI of TR genes. However, it is not well understood how and why TR HI leads to disease. Building upon earlier work (Wilkie 1994; Veitia, 2002; Johnson et al., 2019), we have recently proposed a hypothesis that is based on the crucial role of TR genes in determining and maintaining cell fate and identity (Zug, 2022). We argue (a) that the GRNs that determine cell identity comprise bistable switches, involving positive feedback and cooperativity, (b) that these features make dosage sensitivity of TR genes an inherent property of fate decisions, and (c) that disorders caused by TR HI result from disrupted positive feedback or cooperativity (Zug, 2022).

HI is closely related to the concept of gene essentiality. A gene is defined as essential when loss of its function is associated with a profound loss of fitness; in other words, essential genes do not tolerate loss-of-function variants, and there is strong negative (purifying) selection against those variants (Bartha et al., 2018). Since haploinsufficient genes do not even tolerate the loss of one of the two alleles, HI can be regarded as a particularly strict form of gene essentiality. A recent analysis found that essential genes act predominantly in TR, chromatin modification and lineage specification (Chen et al., 2020), corroborating that developmentally important TR genes tend to be essential.

Box 2: Loss-of-function mutations and their evolutionary implications

Loss of function can result from reduced gene dosage, expression or protein activity (Wilkie, 1994), and be caused by variation in both coding and non-coding sequences:

1. Structural variation (e.g. gene deletions, translocations) can reduce gene dosage.
2. Nonsense and frameshift mutations introduce premature stop codons, which usually results in nonsense-mediated mRNA decay, leading to reduced gene expression.
3. Missense mutations, splice site mutations, and small in-frame indels that inactivate functional protein domains will lead to reduced protein activity.
4. Variation in regulatory sequences can also lead to reduced gene expression via deletion, disruption or disconnection of CREs (so-called regulatory LOF).

The LOF can be complete (gene function eliminated; so-called *amorphic* alleles) or partial (gene function reduced; *hypomorphic* alleles). Hence, any mutation that causes downregulation of a particular gene results in partial LOF.

Loss-of-function mutations have long been regarded as relatively unimportant for adaptive evolution. This is mainly because most LOF mutations are recessive to the wild type and hence phenotypically neutral. However, in haploinsufficient genes, LOF mutations are dominant and thus easily visible to selection, because their effect on fitness is seen in heterozygotes. Therefore, HI counters the argument that LOF mutations cannot play a major role in adaptive evolution (Murray, 2020). In fact, the idea that LOF mutations do contribute to adaptation and evolutionary novelties is gaining momentum (Olson, 1999; Oh et al., 2015; Albalat & Cañestro, 2016; Murray, 2020; Monroe et al., 2021). In the main text, we present evidence supporting this idea with regard to the evolution of human cognitive and social traits, which we argue has been driven by selection for reduced expression (partial LOF) of haploinsufficient TR genes.

Another key finding concerning the genetic architecture of NDDs, which is related to their functional convergence, is the fact that seemingly diverse psychiatric disorders share common risk genes (The Brainstorm Consortium, 2018; Cross-Disorder Group of the Psychiatric Genomics Consortium, 2019; Myers et al., 2020; Lee et al., 2021; Rees et al., 2021). Many of these shared risk genes are involved in TR (Zug, 2022). For example, as described above, HI of *CHD8*, *KMT2C* and *TCF4* is associated with both ASD and SCZ risk. Taken together, many NDD risk genes are involved in TR and characterized by HI and widespread pleiotropy.

Loss of function of haploinsufficient TR genes is also the cause of many NCPs that affect craniofacial development (Table S1). In addition to heterozygous LOF mutations affecting the coding sequences of NCP-associated genes, recent studies have revealed the important role of regulatory LOF in causing NCPs (Laugsch et al., 2019; Long et al., 2020; Sánchez-Gaya et al., 2020). In sum, many NDDs and NCPs are caused by heterozygous LOF of haploinsufficient TR genes through coding or non-coding, regulatory variants of large effect size (modified by common variants of small effect). The fact that many of these TR genes are involved in both brain and face development underscores their pleiotropic nature.

## The role of LOF of TR genes in the evolution of human cognitive and social traits and associated disorders

### Human-specific selection has probably acted on CREs of TR genes whose HI causes NDDs/NCPs

Research into the evolution of human-specific traits has benefited from the increasing availability of genome sequences of other great apes, such as the chimpanzee, and of archaic humans, such as Neanderthals and Denisovans (Pääbo, 2014). These advances have increased our understanding of the genetic peculiarities that distinguish anatomically modern humans (AMHs) from their closest relatives, both living and extinct. For example, comparative genomic analyses have identified hundreds of so-called human accelerated regions (HARs) – genomic loci that are highly conserved among vertebrates yet show accelerated sequence divergence in the human lineage. Importantly, most HARs appear to be driven by positive selection, rather than by non-adaptive processes such as GC-biased gene conversion (Kostka et al., 2012; Hubisz & Pollard, 2014; Levchenko et al., 2018). Moreover, almost all HARs are non-coding CREs (Pollard et al., 2006; Prabhakar et al., 2006; Bird et al., 2007; Lindblad-Toh et al., 2011; Capra et al., 2013; Gittelman et al., 2015), in line with the hypothesis that adaptive divergence in human evolution, particularly with regard to cognitive traits, is primarily driven by regulatory changes (King & Wilson, 1975; Haygood et al., 2010; Enard et al., 2014; Peyrégne et al., 2017; Liu et al., 2021). Strikingly, many HARs have been found to regulate genes that are involved in the TR of brain and face development and for which HI leads to NDDs/NCPs (Table 1; Doan et al., 2018; Levchenko et al., 2018; Kozlenkov et al., 2020; Girskis et al., 2021). Indeed, the vast majority of haploinsufficient TR genes implicated in the evolution of human brain/face development show changes in CREs, rather than coding changes (Table 1). Finally, among 29 human tissues or cell types scanned for signals of positive selection on TF binding sites in CREs (on the basis of predicted binding affinity changes), brain-related cell types show the highest proportion of positive selection (Liu & Robinson-Rechavi, 2020; see also Babbitt et al., 2017). Together, these findings underscore the importance of positive selection on the regulatory architecture of haploinsufficient brain- and face-related TR genes in the evolution of human-specific traits.

**Table 1.** Haploinsufficient transcriptional regulation (TR) genes implicated both in neurodevelopmental disorders (NDDs)/neurocristopathies (NCPs) and in the evolution of human brain and/or face development

TR gene	NDD/NCP owing to haploinsufficiency, including major clinical features related to brain and/or face development^1^	Location/type of human-specific change^2^	References^3^
*ARID1B*	Coffin-Siris syndrome 1 (135900) ID delayed psychomotor development speech and language delay seizures ASD behavioural problems brain anomalies facial dysmorphisms	Differential expression	Berto et al., 2019
*ARID2*	Coffin-Siris syndrome 6 (617808) ID motor delay speech delay ADHD behavioural problems brain anomalies facial dysmorphisms, incl. micrognathia, prominent forehead	Differential expression	Berto et al., 2019
*ASXL1*	Bohring–Opitz syndrome (605039) ID psychomotor retardation seizures brain anomalies microcephaly facial dysmorphisms, incl. micrognathia, prominent forehead	Coding sequence	Dumas et al., 2021
*AUTS2*	Intellectual developmental disorder, autosomal dominant 26 (615834) ID delayed psychomotor development speech delay ASD microcephaly facial dysmorphisms	Coding sequence, CRE (HAR)	Green et al., 2010; Oksenberg et al., 2013; Doan et al., 2016
*BAZ1B*	Major features of Williams–Beuren syndrome (194050) ID poor visual-motor integration poor visual-spatial construction ADHD friendly personality facial dysmorphisms, incl. flat midface	CRE	Kuhlwilm & Boeckx, 2019; Zanella et al., 2019
*CHD8*	Intellectual developmental disorder with autism and macrocephaly (615032) ASD ID seizures macrocephaly facial dysmorphisms	CRE	Moriano & Boeckx, 2020
*CUX1*	Global developmental delay with or without impaired intellectual development (618330) global developmental delay ID	CRE (HAR); differential expression	Doan et al., 2016; Won et al., 2019; Starr et al., 2022
*DNMT3A*	Tatton–Brown–Rahman syndrome (615879) ID ASD seizures brain anomalies macrocephaly facial dysmorphisms	CRE	Castelijns et al., 2020
*EHMT1*	Kleefstra syndrome 1 (610253) ID delayed psychomotor development seizures ASD aggressive behaviour microcephaly facial dysmorphisms	DNA methylation in CRE	Bell et al., 2012
*FOXC1*	Axenfeld–Rieger syndrome, type 3 (602482) cerebellar malformation ocular abnormalities facial dysmorphisms, incl. flat midface	CRE	Prescott et al., 2015
*FOXG1*	Rett syndrome, congenital variant (FOXG1 syndrome; 613454) ID delayed motor development lack of speech development seizures ASD brain anomalies microcephaly	CRE (HAR)	Acosta et al., 2019
*FOXP1*	Intellectual developmental disorder with language impairment and with or without autistic features (613670) ID delayed motor development speech and language deficits ASD ADHD aggression macrocephaly facial dysmorphisms, incl. prominent forehead, retrognathia	CRE (HAR)	Doan et al., 2016
*FOXP2*	Speech language disorder 1 (602081) speech and language deficits brain anomalies	Coding sequence, CRE (HAR)	Enard et al., 2002; Maricic et al., 2013; Caporale et al. 2019; Moriano & Boeckx, 2020; Kozlenkov et al., 2020
*GLI2*	Holoprosencephaly 9 (610829) holoprosencephaly delayed psychomotor development seizures brain anomalies, incl. pituitary hypoplasia microcephaly facial dysmorphisms, incl. flat midface	CRE (HAR)	Won et al., 2019; Norman et al., 2021
*GLI3*	Greig cephalopolysyndactyly syndrome (175700) ID brain anomalies macrocephaly facial dysmorphisms	CRE (HAR)	Won et al., 2019; Moriano & Boeckx, 2020; Hussain et al., 2021
*KDM6A*	Kabuki syndrome 2 (300867) ID speech delay motor delay seizures behavioural difficulties brain anomalies facial dysmorphisms	CRE	Moriano & Boeckx, 2020
*MEF2C*	Neurodevelopmental disorder with hypotonia, stereotypic hand movements, and impaired language (613443) ID lack of speech development delayed motor development seizures ASD brain anomalies facial dysmorphisms	CRE (HAR)	Doan et al., 2016
*NFIB*	Macrocephaly, acquired, with impaired intellectual development (618286) ID ASD ADHD behavioural anomalies, incl. aggression, anxiety brain anomalies macrocephaly facial dysmorphisms	CRE	Moriano & Boeckx, 2020
*NFIX*	Malan syndrome (614753) ID speech delay motor retardation ASD behavioural anomalies, incl. aggression, anxiety brain anomalies macrocephaly facial dysmorphisms	DNA methylation in CRE	Gokhman et al., 2020
*NPAS3*	Disorder as yet unnamed ID SCZ BD	CRE (HAR)	Kamm et al., 2013
*NR2F2*	Congenital heart defects, multiple types, 4 (615779) developmental delay facial dysmorphisms	CRE (HAR)	Doan et al., 2016
*PAX3*	Waardenburg syndrome, type 1 (193500) facial dysmorphisms skin hypopigmentation	CRE	Prescott et al., 2015
*PAX6*	Aniridia 1 (106210) brain anomalies facial dysmorphisms	CRE (HAR)	Won et al., 2019
*PITX2*	Axenfeld–Rieger syndrome, type 1 (180500) facial dysmorphisms	CRE	Prescott et al., 2015
*POGZ*	White–Sutton syndrome (616364) ID delayed psychomotor development ASD behavioural problems microcephaly facial dysmorphisms, incl. midface hypoplasia	Coding sequence	Green et al., 2010
*POU3F2*	Disorder as yet unnamed developmental delay ID ASD SCZ BD facial dysmorphisms	CRE (HAR)	Gittelman et al., 2015; Won et al., 2019
*POU3F3*	Snijders Blok–Fisher syndrome (618604) developmental delay ID speech and language delay seizures ASD behavioural abnormalities brain anomalies facial dysmorphisms	CRE (HAR)	Prescott et al., 2015; Won et al., 2019
*RORB*	Epilepsy, idiopathic generalized, susceptibility to, 15 (618357) seizures developmental delay ID speech delay ASD behavioural problems, incl. aggression	CRE (HAR)	Won et al., 2019
*RUNX2*	Cleidocranial dysplasia (119600) skull anomalies facial dysmorphisms, incl. midface hypoplasia, micrognathia	Coding sequence, CRE (HAR)	Green et al., 2010; Gittelman et al., 2015; Di Pietro et al., 2021
*SATB2*	Glass syndrome (612313) ID delayed psychomotor development poor speech development seizures behavioural problems, incl. aggression happy demeanor microcephaly facial dysmorphisms, incl. high forehead, midface hypoplasia, micrognathia	CRE	Weiss et al., 2021
*SETD1A*	Neurodevelopmental disorder with speech impairment and dysmorphic facies (619056) ID speech delay, impaired language motor delay seizures ASD SCZ behavioural problems, incl. aggression, anxiety overfriendliness facial dysmorphisms, incl high forehead	CRE	Moriano & Boeckx, 2020
*SOX2*	Microphthalmia, syndromic 3 (206900) ID psychomotor delay brain anomalies, incl. pituitary hypoplasia microcephaly facial dysmorphisms	CRE (HAR)	Won et al., 2019
*SOX6*	Tolchin–Le Caignec syndrome (618971) ID ASD ADHD behavioural problems, incl. aggression, anxiety craniosynostosis facial dysmorphisms, incl. high forehead, micrognathia	CRE (HAR)	Gittelman et al., 2015
*SOX9*	Campomelic dysplasia (114290) brain anomalies macrocephaly facial dysmorphisms, incl. high forehead, flat face, micrognathia	DNA methylation in CRE	Gokhman et al., 2020
*TBR1*	Intellectual developmental disorder with autism and speech delay (606053) ID developmental delay delayed speech/absence of speech delayed walking ASD brain anomalies	CRE (HAR)	Won et al., 2019
*TCF4*	Pitt–Hopkins syndrome (610954) ID poor or absent speech development delayed motor development seizures ASD behavioural problems, incl. aggression happy personality brain anomalies microcephaly facial dysmorphisms	CRE	Mozzi et al., 2017; Moriano & Boeckx, 2020
*TCF20*	Developmental delay with variable intellectual impairment and behavioural abnormalities (618430) ID motor delay speech delay seizures ASD ADHD behavioural anomalies, incl. aggression, anxiety brain anomalies macrocephaly facial dysmorphisms, incl. midface hypoplasia, prominent forehead	Differential expression	Berto et al., 2019
*TWIST1*	Saethre–Chotzen syndrome (101400) craniosynostosis facial dysmorphisms, incl. high forehead, flat face, maxillary hypoplasia	? (Transcriptome sequencing)	Sousa et al., 2017b
*ZBTB20*	Primrose syndrome (259050) ID ASD behavioural problems, incl. aggression, anxiety brain anomalies macrocephaly facial dysmorphisms, incl. midface hypoplasia	Coding sequence	Green et al., 2010
*ZEB2*	Mowat–Wilson syndrome (235730) ID delayed motor development impaired or absent speech seizures behavioural problems, incl. repetitive behaviours happy demeanor brain abnormalities microcephaly facial dysmorphisms	CRE (HAR)	Erwin et al., 2014

Notes: 1, If available, the OMIM phenotype number is given in parentheses. Listed clinical features do not necessarily appear in all (or even most) patients. ADHD, attention deficit hyperactivity disorder; ASD, autism spectrum disorder; BD, bipolar disorder; ID, intellectual disability; NCP, neurocristopathy; NDD, neurodevelopmental disorder; SCZ, schizophrenia. 2, Changes occurred in the evolutionary lineage leading to anatomically modern humans (AMHs), either compared with archaic humans or with non-human primates. CRE, *cis-*regulatory element; HAR, human accelerated region. 3, References for human-specific evolutionary changes of the particular gene. For references for the NDD/NCP caused by haploinsufficiency of the gene, see Table S1 in the Supplementary Material.

### Evidence of reduced TR gene expression during human evolution

**Figure 2.**
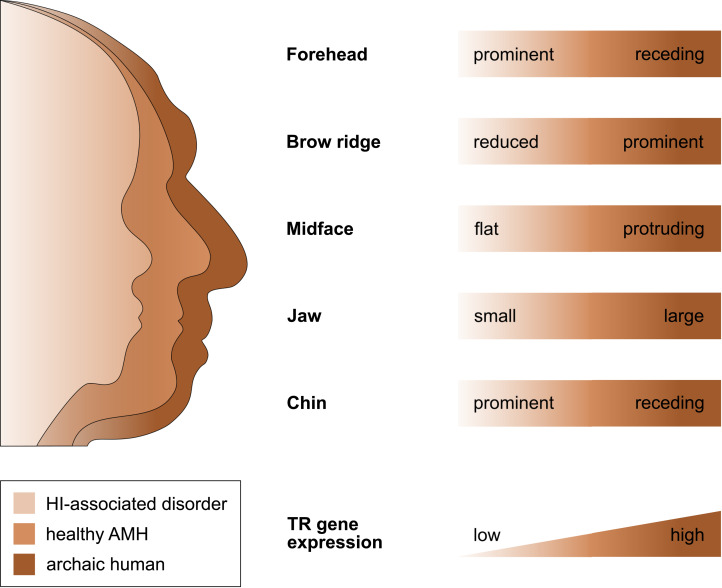
The parallelism between the evolutionary and the pathological consequences of reduced TR gene expression for craniofacial development. The figure shows several craniofacial features of archaic humans, healthy anatomically modern humans (AMHs), and AMHs with a haploinsufficiency-associated disorder. Both the evolutionary changes from archaic humans to AMHs and the pathological changes from healthy AMHs to individuals with the disorder are due to reduced TR gene expression. Adapted from Gokhman et al. (2020).

That selection appears to have acted predominantly on CREs, and that the TR genes regulated by those CREs are exquisitely dosage sensitive (as evidenced by their HI), suggests that the associated human-specific traits evolved, at least in part, owing to changes in the genes’ expression level. Indeed, with respect to craniofacial traits, there is good support for this idea, in particular, for a *reduction* in gene expression (i.e. partial LOF). As described in detail below, several studies indicate (1) that the phenotypic changes in craniofacial morphology that occurred from archaic to modern humans are due to reduced TR gene expression and (2) that these phenotypic changes seen during human evolution resemble the phenotypic changes seen in HI-associated disorders, which are likewise due to reduced TR gene expression. In other words, there seems to be a parallelism between the evolutionary and the pathological consequences of reduced TR gene expression for craniofacial features (Figure 2). For example, reduced expression of the haploinsufficient TF gene *NFIX* causes Malan syndrome, whose clinical features include a high forehead and a prominent chin (Priolo et al., 2018). Intriguingly, the same genetic and phenotypic changes (reduced *NFIX* expression, resulting in a more prominent forehead and chin) are likely to have also occurred in the evolution from archaic to modern humans (Gokhman et al., 2020). A similar correspondence between the evolutionary and the pathological changes in craniofacial traits following TR gene downregulation has been suggested for *BAZ1B* (reduced facial bones; Williams–Beuren syndrome; Zanella et al., 2019), and *SATB2* (smaller jaws, flatter face, higher forehead; SATB2-associated syndrome; Weiss et al., 2021); both cases are discussed in more detail in the next section. Taken together, these findings suggest that downregulation of haploinsufficient TR genes might have played an important role in the evolution of human craniofacial traits.

What do we know about TR gene expression changes in the evolution of the human brain? A promising approach to address this question is to compare gene expression patterns in brain samples from humans and other primates. In the past few years, it has become possible to compare brain transcriptomes at single-cell resolution, which enables the detection of cell type-specific gene expression differences. Interestingly, comparisons between primates show that, in the human lineage, gene expression divergence is higher in non-neuronal cell types such as oligodendrocytes than in neurons (Berto et al., 2019; Hu et al., 2020; Khrameeva et al., 2020; Pembroke et al., 2021). Strikingly, genes that showed human-specific downregulation in oligodendrocytes were enriched for functions related to TR; among those downregulated TR genes were *ARID1B*, *ARID2* and *TCF20* (Berto et al., 2019). Other brain-related TR genes that probably experienced reduced expression during human evolution are *FOXP2* and *CUX1*. Maricic et al. (2013) found a regulatory change in *FOXP2* specific to modern humans that diminishes binding of the TF POU3F2, thus driving reduced reporter gene expression compared with the ancestral allele. Finally, using allele-specific expression data from human–chimpanzee hybrid cortical spheroids, Starr et al. (2022) recently found that expression of *CUX1* from the human allele is lower than expression from the chimpanzee allele. These results suggest that reduced TR gene expression has played a role in human brain evolution. We do not know the specific effects that downregulation of these TR genes had on human brain development. However, this knowledge is crucial to understand how reduced expression affected the evolution of human cognition and social behaviour. Therefore, we cannot yet determine the relationship between the evolutionary and the pathological consequences of reduced TR gene expression for brain-related traits (remember that, for craniofacial traits, there is tentative evidence of a parallel relationship). The fact that TR HI in NDDs generally leads to ID and other impairments of brain function (Table 1) argues against a simple parallel relationship between the pathological and the evolutionary consequences of reduced TR gene expression with respect to brain-related traits, because cognitive function has increased, rather than decreased, during human evolution.

While few TR genes have so far been studied in detail, the findings described above suggest that reduced TR gene expression was involved in the evolution of both human craniofacial and brain-related traits. Thus, the results also add to the long-standing discussion about the developmental mechanisms that contributed to the evolution of human-specific traits. While many researchers have argued for a major role of heterochrony (or more specifically, delayed gene expression) in human evolution (Gould, 1977; Somel et al., 2009), the findings presented here emphasize the role of *reduced* gene expression (i.e. heterometry; see Arthur, 2000). Nevertheless, the conclusion that reduced TR gene expression was important during human evolution does not preclude the possibility that, in other developmental contexts, it was *increased* TR gene expression that fuelled the evolution of human-specific traits. Moreover, the effects of TR gene expression changes on development and evolution of craniofacial traits are not necessarily the same as those on development and evolution of brain-related traits (see Box 3). Also, our conclusion is currently based only on a handful of cases and hence somewhat speculative, while several crucial questions remain open. Nevertheless, we think that HI of TR genes predisposes these genes to LOF mutations that do have evolutionary implications (see Box 2). Therefore, we believe that future research will unearth further evidence supporting the significance of TR gene downregulation in the evolution of human-specific traits.

Box 3: Evolution and development of human brain and face – evidence of partial independence between both structures

Modern humans have a large, globular brain and a short, retracted face that distinguish them from their extinct *Homo* relatives. Based on recent morphometric analyses of *Homo sapiens* fossils, the evolution of modern human brain and face can be roughly divided into an early and a later stage. Hominin fossils from around 300,000 years ago suggest that early *H. sapiens* already showed key features of modern human craniofacial morphology (Hublin et al., 2017; Lacruz et al., 2019). Brain sizes in early *H. sapiens* also already fell within the range of those of present-day humans. In contrast, brain shape changed only considerably later, resulting in the typical globular modern shape between about 100,000 and 35,000 years ago. Intriguingly, this later stage involving brain globularization parallels the emergence of behavioural modernity, the suite of behavioural and cognitive traits that distinguishes modern humans from other hominins (Neubauer et al., 2018). It seems likely that the emergence of these traits in modern humans was facilitated by the two major features contributing to brain globularization: bulging of the parietal cortex and of the cerebellum (Boeckx, 2017; Kochiyama et al., 2018; Neubauer et al., 2018; Pereira-Pedro et al., 2020). Both brain areas are engaged in a broad range of sensorimotor, cognitive and social functions (Sokolov et al., 2017; Freedman & Ibos, 2018). Hence, parietal and cerebellar bulging seem to be particularly important for the emergence of behavioural modernity.

These findings suggest that modern human cognition and behaviour evolved later than, and partially independently from, the modern human face. Indeed, this partial independence of human brain and face becomes apparent not only during evolution, but also in development. Naqvi et al. (2021) searched for genomic loci that influence both human face and brain shape and found that many of the shared brain-face loci include TR genes involved in craniofacial development. However, they also found that only a subset of shared loci indeed affects behavioural–cognitive traits and neuropsychiatric disorders. For example, shared TR genes that are involved in both NC and brain development (e.g. *TCF4*, *ZEB2*) do affect behavioural–cognitive and neuropsychiatric traits, while other shared TR genes are only involved in NC development, with no effects on brain-related traits (e.g. *PAX3*, *TWIST1*). Hence, genes affecting craniofacial traits are not necessarily the same as those affecting cognitive and behavioural traits.

### Selection for prosociality and against fear and aggression: the human self-domestication hypothesis

In light of the evidence of reduced TR gene expression during human evolution, an important question is: what selective forces were responsible for this downregulation and the associated emergence of human-specific traits? One hypothesis is that these traits evolved in part through human self-domestication (HSD) (Hare, 2017; strictly speaking, the term is a misnomer – in contrast to animal domestication, HSD did not involve any *deliberate* selection or goal-directedness; Sánchez-Villagra & van Schaik, 2019). According to the HSD hypothesis, selection during human evolution favoured in-group prosociality over aggression and fear. In the wake of this selection, humans evolved traits similar to those characteristic of other domestic animals. In domesticated mammals, this suite of behavioural, physiological and morphological traits that emerged via selection for tameness is referred to as the ‘domestication syndrome’ (Wilkins et al., 2014). Several craniofacial traits that distinguish modern humans from their archaic ancestors, such as a short, retracted face and a reduced brow ridge (Figure 2), fit the domestication syndrome, which can be seen as support for the HSD hypothesis (Hare, 2017). In their seminal paper, Wilkins et al. (2014) propose that the domestication syndrome ultimately results from multiple partial LOF mutations affecting dosage-sensitive NC genes (the ‘neural crest/domestication syndrome’ hypothesis; for an up-to-date account, see Wilkins et al., 2021). Indeed, many haploinsufficient TR genes involved in NC development, including *BAZ1B*, *CHD7*, *MITF*, *PAX3*, *PAX9*, *RAI1*, *SATB2*, *SOX9*, *SOX10* and *ZEB2*, are not only known to cause human NCPs (Table S1), but also are candidate genes for the domestication syndrome (Wilkins et al., 2014; Pendleton et al., 2018). Hence, the neural crest/domestication syndrome hypothesis provides an appealing mechanism underlying HSD. Yet what is the evidence for this hypothesis with respect to human evolution?

In order to test the neural crest/domestication syndrome hypothesis with regard to HSD, Zanella et al. (2019) focused on Williams–Beuren syndrome, whose characteristic features include a reduced, retracted face and pronounced friendliness and sociality – traits that fit the domestication syndrome. HI of the chromatin regulator *BAZ1B* has long been known as the causal factor for most of the characteristic features of Williams–Beuren syndrome (Lu et al., 1998; Peoples et al., 1998; Lalli et al., 2016). Zanella et al. (2019) showed that *BAZ1B* is a master regulator of NC genes involved in craniofacial development, and Kuhlwilm and Boeckx (2019) found nearly fixed mutations in the CREs of *BAZ1B* in the genomes of modern, but not archaic, humans. These mutations are probably partial LOF mutations causing slight reductions in gene expression and, ultimately, in craniofacial morphology, corresponding to the facial retraction from archaic to modern humans (Wilkins, 2020). Strikingly, very similar findings were recently described for another NC gene, the TF *SATB2*. HI of *SATB2* causes SATB2-associated syndrome (also termed Glass syndrome), whose features include small jaws, flat face and a friendly personality – again, traits that match the domestication syndrome. Weiss et al. (2021) found a fixed mutation in the CRE of *SATB2* in modern, but not archaic, humans, which probably reduced *SATB2* expression and contributed to the retracted face characteristic of modern humans. In light of the correspondence between the pathological and the evolutionary changes in craniofacial traits following reduced TR gene expression (see above), these findings provide tentative support for the neural crest/domestication syndrome hypothesis as a mechanism underlying HSD: selection for prosociality led to reduced expression of cranial NC-associated genes (e.g. *BAZ1B*, *SATB2*), and this NC hypofunction caused, pleiotropically, the craniofacial changes that distinguish modern humans (and which are paralleled both in the associated HI syndromes and in the domestication syndrome of domesticated mammals). Moreover, the neural crest/domestication syndrome hypothesis as a possible mechanism for HSD has also been linked to the evolution of human cognition and language, based on a set of genes (many of them encoding TRs) that are involved both in NC development and in brain globularization (Benítez-Burraco et al., 2018; Thomas & Kirby, 2018).

Given the appeal of the HSD hypothesis as an explanation for the emergence of human cognitive and social traits, it is not surprising that researchers have used it to understand the origin and persistence of psychiatric disorders. SCZ, for example, is viewed as a hyper-domestication syndrome, resulting from NC hypofunction and hence from the same selective pressures that triggered HSD (Benítez-Burraco et al., 2017; Šimić et al., 2021). In fact, this idea is only one of several evolutionary hypotheses that trace the existence of psychiatric disorders to the action of natural selection. In contrast, in the next section, we argue that the important role of LOF of TR genes in the etiology of psychiatric disorders is more compatible with the view that these disorders persist despite, not because of, natural selection.

### Evolutionary models for the persistence of psychiatric disorders

NDDs such as ASD and SCZ seem to present an evolutionary paradox: they have a strong genetic component and are associated with considerable fitness costs, and yet they are surprisingly prevalent. Given the high heritability and high fitness costs of these disorders, why has natural selection not eliminated the genetic variants that predispose to them? This ‘paradox of common, harmful, heritable mental disorders’ (Keller & Miller, 2006) has long puzzled evolutionary geneticists and psychiatrists alike (e.g. Huxley et al., 1964). In essence, it boils down to a fundamental question in evolutionary genetics: what causes genetic variation in fitness-related traits to persist, given that selection is expected to minimize it?

Simply put, there are two classes of evolutionary models to explain genetic variation in fitness-related traits and, by extension, in the liability to psychiatric disorders: mutation-selection balance (or more precisely, mutation-selection-drift) and balancing selection (Keller, 2018). The mutation-selection-drift model explains genetic variation as a balance between the loss of deleterious alleles through purifying selection and the emergence of new ones through mutation; in addition, random events cause some deleterious alleles with small effects to drift to high frequencies. Under the alternative model, balancing selection, allelic variation at a given locus is maintained by the balance between positive and negative selective forces. In this scenario, risk alleles associated with psychiatric disorders confer some benefit at least under some conditions; hence, selection for the beneficial trait can maintain disorder risk as a by-product. For example, Crow (1997) famously argued that SCZ was ‘the price that *Homo sapiens* pays for language’. Indeed, the idea that SCZ represents a maladaptive by-product of positive selection during human evolution has been expressed repeatedly (Burns, 2004; Crespi et al., 2007; Sikela & Searles Quick, 2018), and the HSD approach to SCZ is one version of this idea (see above). Similar hypotheses have been suggested for ASD (Baron-Cohen, 2003; Ploeger & Galis, 2011) and bipolar disorder (Wilson, 1998; Greenwood, 2020). A major difference between mutation-selection balance and balancing selection concerns the role of selection: under mutation-selection balance, mental disorders exist and persist *despite* natural selection, whereas under balancing selection, they do so *because of* natural selection (Durisko et al., 2016). Which model, then, best explains the paradox of harmful yet common psychiatric disorders?

As our understanding of the genetic architecture of ASD, SCZ and other highly heritable psychiatric disorders is increasing, much research is consistent with a simple mutation-selection-drift model (Keller & Miller, 2006; Keller, 2018). First, there is broad support for the important role that rare deleterious mutations with large effect size play in the etiology of NDDs: as described above, a considerable part of ASD and SCZ risk is due to rare heterozygous LOF variants in TR genes that do not tolerate such LOF because of their HI. In addition, disorder risk also depends on a large number of deleterious variants with small effect sizes (background polygenic risk). In fact, the effects of many deleterious mutations will be so small that they may drift to high frequencies. Since the extremely polygenic nature of complex traits (Boyle et al., 2017) implies a large mutational target size of psychiatric disorders, this helps to explain their high prevalence (Keller, 2018). Second, there is compelling evidence that, on average, risk alleles associated with these disorders are under weak to strong purifying selection (Rees et al., 2011; Mullins et al., 2017; Keller, 2018; Pardiñas et al., 2018; Huang & Siepel, 2019; Esteller-Cucala et al., 2020; Rapaport et al., 2021; Wendt et al., 2021). These lines of evidence converge in recent population genetic analyses which apply a simple mutation-selection balance model (either as a deterministic approximation or explicitly incorporating drift) to provide direct estimates of the strength of selection against heterozygous LOF in humans (Cassa et al., 2017; Weghorn et al., 2019; Agarwal et al., 2022). Strikingly, negative selection was strongest for LOF-intolerant TR genes (Cassa et al., 2017).

Recent studies provide further interesting insights into the nature of negative selection at LOF-intolerant genes. First, purifying selection not only acts on coding sequences, but also on the promoters of LOF-intolerant genes, presumably to safeguard promoter hypomethylation and to curb coding mutation rates (Boukas et al., 2022). Second, negative selection at LOF-intolerant genes comes not only in the form of natural selection (against variants increasing pre-reproductive mortality or decreasing fertility), but also in the form of sexual selection. Gardner et al. (2022) found that LOF variants in these genes reduce reproductive success much more in males than in females, and that this reduction is mediated primarily through cognitive and behavioural traits, rendering male carriers of such variants less likely to find mating partners (see also Liu et al., 2022).

Lastly, we would like to add two further points. First, while mutation-selection-drift offers a convincing explanation for the persistence of highly heritable psychiatric disorders such as ASD and SCZ, other NDDs, and especially those with lower heritability, might require different types of evolutionary explanation. For example, many symptoms of depression, which has much lower heritability than other psychiatric disorders, can be seen as adaptive defences to minimize fitness loss in the face of adverse life situations (Keller, 2018; Syme & Hagen, 2020). Second, acceptance of mutation-selection-drift as the major explanation of the *persistence* of psychiatric disorders does not preclude a role of positive selection in providing the conditions for the evolutionary *origin* of these disorders. One can easily imagine a scenario in which mutations that altered brain development of early *H. sapiens* were selected for, became fixed, and thus helped to establish human-specific cognitive and behavioural abilities (e.g. mutations that gave rise to HARs). At the same time, these mutations could have, if only indirectly, made human brain development more susceptible to disruption and allowed disorders such as ASD and SCZ to arise (we thank an anonymous reviewer for drawing our attention to such a scenario). However, since these mutations became fixed early on in human evolution, such a scenario does not explain individual differences in genetic risk to psychiatric disorders in modern human populations (Keller, 2018).

Taken together, positive selection might have been involved in the emergence of psychiatric disorders, and we also cannot completely rule out balancing selection as a possible explanation for the persistence of some risk alleles associated with these disorders. Nevertheless, mutation-selection-drift is likely to be a more general explanation for the observed genetic variation in disorder risk (Keller, 2018). Both the evidence for disease-causing LOF alleles in LOF-intolerant TR genes and for strong purifying selection against such alleles are fully in line with this conclusion.

## Predictions and suggestions for future work

Based on the evidence discussed in this article, we hypothesize that human-specific cognitive and social traits evolved due to reduced expression (partial LOF) of haploinsufficient TR genes, possibly through selection for lower aggressiveness and higher sociality. Although it is difficult to test this hypothesis directly, there are several ways to obtain indirect evidence. For example, comparative functional genomics provides a powerful tool to investigate the evolutionary differences in brain and face development between humans and their closest relatives, both living and extinct, thus advancing our understanding of the molecular basis of uniquely human traits (Konopka et al., 2012; Enard, 2016; Reilly & Noonan, 2016; Kuhlwilm & Boeckx, 2019). As described above, such comparative studies have already found evidence of reduced expression of TR genes (*ARID1B*, *ARID2*, *BAZ1B*, *CUX1*, *FOXP2*, *NFIX*, *SATB2*, *TCF20*) in modern, but not archaic, humans, some of which probably contributed to the retracted face characteristic of present-day humans (Zanella et al., 2019; Gokhman et al., 2020; Weiss et al., 2021). In this vein, comparative genomics is likely to provide further evidence of partial LOF of haploinsufficient TR genes as an evolutionary driver of human-specific brain and craniofacial traits.

A particularly powerful experimental model system that has recently emerged to tackle previously intractable questions of human evolution are brain organoids generated from induced pluripotent stem cells (iPSCs) (Dannemann & Gallego Romero, 2022; Mora-Bermúdez et al., 2022). For example, Benito-Kwiecinski et al. (2021) used brain organoids to show that heterozygous LOF of *ZEB2* results in an enlarged neuroepithelium, consistent with brain expansion during human evolution. Hence, future work could study the effects of LOF of other brain-related TR genes in human brain organoids and compare them with organoids derived from other hominids, including both archaic humans and non-human primates. Another recently developed technique to study gene regulatory divergence in the evolution of human brain and face development involves fusing human and chimpanzee iPSCs to generate interspecies hybrids (Agoglia et al., 2021; Gokhman et al., 2021). Since in hybrids both alleles experience the same environment, including *trans-*acting regulators, this approach is particularly well-suited for identifying *cis-*regulatory changes between species. Recently, this method was used to detect reduced *CUX1* expression in humans (Starr et al., 2022), and we expect future studies using interspecies hybrids to reveal further examples of human-specific TR gene downregulation.

The selective forces that drove the evolution of human cognition and sociality are not amenable to direct testing. However, if the HSD hypothesis has some merit, then a comparative approach involving domesticated animal species should provide insights. The hypothesis suggests that the evolution of domesticated species *and* modern humans was driven by selection against fear and aggression and for tameness/prosociality. For example, humans and domestic dogs have evolved similar social cognitive skills (in both cases adapted for social and communicative interactions with human beings), indicative of convergent evolution (Hare & Tomasello, 2005). Therefore, comparative genomics across different domesticated species can increase our understanding of the selective conditions that promoted the evolution of human cognition and sociality. For example, genomic analyses of several domesticated species, including humans, found signs of positive selection in NC-related genes, in line with both the neural crest/domestication syndrome hypothesis and the HSD hypothesis (Theofanopoulou et al., 2017; Pendleton et al., 2018). Specifically, we predict that NC-related TR genes in domesticated species exhibit reduced expression (partial LOF) compared with their wild ancestors (see also Wilkins et al., 2021). In light of the convergent evolutionary processes among domesticated species and modern humans, such findings would lend further support to the idea that human cognitive and social traits evolved (at least in part) in response to selection for prosociality and against fear and aggression.

## Conclusions

Clearly, the question of what makes us human can be approached from different angles (Varki et al., 2008; Calcagno & Fuentes, 2012). In this article, we focus on the evolution of human cognition and sociality and approach it from a TR perspective. In particular, we argue that loss of function of genes involved in TR of brain and face development is crucial to understand both the evolution and dysfunction of our unique cognitive and social skills. It is now possible for comparative and experimental studies to put the theory to the test.

## Data Availability

N/A

## Research transparency and reproducibility

N/A

## Supplementary material

For supplementary material accompanying this paper visit https://doi.org/10.1017/ehs.2022.42. click here to view supplementary material
